# Nutritional evaluation of some potential wild edible plants of North Eastern region of India

**DOI:** 10.3389/fnut.2023.1052086

**Published:** 2023-03-01

**Authors:** Hammylliende Talang, Aabon Yanthan, Ranbir Singh Rathi, Kanakasabapathi Pradheep, Soyimchiten Longkumer, Bendangla Imsong, Laishram Hemanta Singh, Ruth S. Assumi, M. Bilashini Devi, Vanlalruati, Ashok Kumar, Sudhir Pal Ahlawat, Kailash C. Bhatt, Rakesh Bhardwaj

**Affiliations:** ^1^ICAR Research Complex for NEH Region, Umiam, Meghalaya, India; ^2^ICAR Research Complex for NEH Region, Nagaland Centre, Medziphema, Nagaland, India; ^3^ICAR-National Bureau of Plant Genetic Resources, New Delhi, India; ^4^School of Agricultural Sciences and Rural Development, Medziphema, Nagaland, India

**Keywords:** proximate composition, minerals, antioxidants, ethnic foods, biodiversity

## Abstract

**Introduction:**

India’s north-eastern hill region (NEH) is one of the biodiversity hotspots, inhabited by several tribal communities still maintaining their traditional food habits. Much of their food resources are drawn from wild sources.

**Materials and methods:**

Fourteen species of wild edible plants of high ethnic importance were collected from remote localities of Nagaland and Meghalaya states of the NEH region of India for nutritional profiling. Nutritional profiling of leaves of six species comprising *Gynura cusimbua, Garcinia cowa, Herpetospermum operculatum, Plukenetia corniculata, Trichodesma khasianum*, and *Elatostemma sessile* is conducted first time under present study. Samples were analyzed as per the Official Method of Analysis (AOAC) and standard methods.

**Results and discussion:**

The range of variation in proximate composition was observed for moisture (72–92%), protein (1.71–6.66%), fat (0.22–1.36%), dietary fibre (5.16–14.58%), sugar (0.30–3.41%), and starch (0.07–2.14%). The highest protein content (6.66%) was recorded in *Herpetospermum operculatum*, followed by *Trichodesma khasianum* (5.89%) and *Plukenetia corniculata* (5.27%). Incidentally, two of these also have high iron (>7.0 mg/100 g) and high zinc (>2.0 mg/100 g) contents, except *Trichodesma khasianum*, which has low zinc content. High antioxidant activities in terms of gallic acid equivalent (GAE) by the cupric ion reducing antioxidant capacity (CUPRAC) method ranged from 1.10 to 8.40 mg/100 g, and by the Fluorescence recovery after photobleaching (FRAP) method ranged from 0.10 to 1.9 mg/100 g, while phenol content ranged between 0.30 and 6.00 mg/100 g. These wild vegetables have high potential because of their nutritional properties and are fully capable of enhancing sustainability and improving ecosystem services. Efforts were also initiated to mainstream these resources, mainly for widening the food basket of native peoples.

## 1. Introduction

The north-eastern hill (NEH) region of India comprising the states of Arunachal Pradesh, Assam, Manipur, Meghalaya, Mizoram, Nagaland, Tripura, and Sikkim lies between 21°50’ and 29°34’N latitude and 85°34’ and 97°50’E longitude, is one of the biodiversity hotspots of the world. The region’s diverse topography, altitude, soil, and climate are favorable for the evolution and development of different wild species (1). About 800 different species of wild edible plants have been reported in India. Around 300 species are utilized by the tribal population of the NEH region for food and medicinal purposes (2–5). Generally, these plants grow in wild and semi-wild conditions, are less known to the mass population, and have lesser market demand.

Wild edible plants (WEPs’) are an essential component of ethnic foods. They are integrated with the culture and tradition of many indigenous communities worldwide, serving as a staple food for indigenous people and as a supplement to non-indigenous people’s diets (6). They are a rich source of micro-nutrients used in treating several diseases (7). When analyzed for nutritional value, many WEPs have more vitamins (A and C) and folic acid and exhibit remarkable antioxidant properties than common vegetables (8–10).

Inventory of such wild food resources, associated indigenous knowledge, mode of usage, and present status, coupled with nutritional evaluation, can establish an alternative to achieve food and nutritional security up to a large extent. Recently, the demand for healthy food and increasing health consciousness among the masses has created renewed global interest in utilizing wild food plants. Efforts were also initiated to mainstream these resources, mainly for widening the food basket of native peoples (8).

In NEH region, the inhabitants live in and around forest areas, and practice shifting cultivation, where several crops are cultivated. Besides, inhabitants gather wild edible plants from community forests and some are also grown in kitchen gardens/homesteads. These communities follow traditional processing and cooking methods to make them palatable and suitable for consumption by children, adults, pregnant and lactating women, and ill persons using traditional wisdom. The region is also known for its diverse food basket, which is also indicated in dietary diversity, an indicator of adequate micronutrients in food intake. In the national family health survey (NFHS-5) India survey 2019–2021, the criteria of feeding the child food from at least four food groups were considered as minimum dietary diversity (MDD). Results on MDD have shown contrasting values for the NEH region and the rest of India. NEH region of India has 38% of youngest children aged 6–23 months living with their mother meeting MDD, while the average value for India is 24.1%. This is also indicated in other health parameters particularly the prevalence of any anemia (hemoglobin < 11.0 g/dl) in children of 06–59 months where 67.1% are anemic in India. However, rich dietary diversity has contributed to lower incidences (50.6%) in the NEH region. Similarly, among women of age, 15–49 years country average is 57.0% whereas for the NEH region is 51.9 (11).

NFHS-5 findings are also an indicator of the role and nutritional potential of wild edible plants, however, that cannot be accounted for in dietary intake due to the lack of nutrient composition data for these resources. Thus an attempt has been made to record their edible value and estimate their nutritional properties. This study will help assess these WEPs contribution to nutrient intake and determine their potential to improve the socio-economic status of local communities in the NEH region.

This study provides robust data for important wild edible plants of the NEH region, which were collected thrice during 2016–2019 on edible proportion, proximate composition, total sugars, total starch, total phenols, antioxidant potential, and important minerals. However, in this study, we could not include the estimation of vitamins, oxalates, and profiles for polyphenols, amino acids, fatty acids, phytosterols, sugars, and oligosaccharides.

## 2. Materials and methods

### 2.1. Documenting traditional knowledge (TK) on wild edible plants (WEPs)

For gathering information on indigenous technical knowledge which includes local names, uses, parts used and methods of processing wild edible plants, experienced elderly persons were selected as respondents and in the presence of local guides, they were asked open-ended questions in the local dialect (12). Questions in the interview schedule include traditional knowledge (TK) about local edible plant species, the habitat of edible plant species, the economic value of species, and cultural and livelihood dimensions of the foods. Based on the responses from the respondents, indigenous traditional knowledge on wild edible plants available in the study areas was collected and documented.

### 2.2. Collection and identification of plants samples

A total of 10 field surveys were carried out in parts of Nagaland and Meghalaya states four each in 2016 and 2018 and two in 2019 in different seasons to identify, collect and document the local plant species used in the tribal foods of North Eastern Hill (NEH) region. Based on the inhabitants’ information and consulting regional floras and expert taxonomists based at NBPGR, New Delhi and Botanical Survey of India, Eastern Circle, Shillong, fourteen wild edible plants, namely, *Gynura cusimbua, Centella asiatica, Diplazium esculentum, Garcinia cowa, Eryngium foetidum, Zanthoxylum rhetsa, Houttuynia cordata*, *Clerodendrum glandulosum, Herpetospermum operculatum, Plukenetia corniculata, Trichodesma khasianum, Piper pedicellatum, Litsea cubeba, Elatostemma* sp., were identified and prioritized for their nutritional profiling.

### 2.3. Analysis of samples for proximate composition

The samples were washed initially with tap water to remove any adhering substances, followed by double distilled water, while surface water was removed by spreading evenly under a fan. Finally, the samples were evaluated in duplicate by following AOAC (13); moisture (934.01), ash (938.08), crude fat (920.58) by gravimetric method; total dietary fiber (985.29) enzymatic gravimetric method; total protein (2011.11) by kjeldahl method using jones factor 6.25, total starch (996.11) enzymatic colorimetric assay, and mineral profiles by atomic absorption spectrometry (AAS, 999.11). The total soluble sugars (14), total phenols (15), ferric-reducing antioxidant potential (16), and cupric-reducing antioxidant capacity (17) were analyzed as explained in Singh et al. (8), including method validation to ensure data quality. Results were analyzed for bivariate Pearson correlation using SPSS17.

## 3. Results and discussion

Traditional communities of the NEH region have considerable dependence on wild edible plants and have valued them based on experiential learning for food and medicinal value. In this study, we have presented only those claims w.r.t. medicinal value which is part of common knowledge in multiple communities, confirmed by traditional healers, and supported through published literature. Wide variation was observed in the nutritional value of these resources such as in the content of protein 1.6–6.7%, iron 1.86–9.4 mg/100 g, total phenols, and antioxidant potential. Results from this study are discussed below.

### 3.1. Traditional knowledge of wild edible plants

Many indigenous communities worldwide rely on wild edible plants as part of their diet (18, 19), and as a potential source of bioactive compounds, colors, and flavors (20). Traditional knowledge on the recipe, food, and medicinal uses was recorded from the tribal people of Nagaland and Meghalaya. The uses and method of processing, botanical name, family, growth habit, habitat, flowering and fruiting season, and edible parts of selected plants under the study are presented in Supplementary Table 1. All fourteen plants are used locally for vegetables, salad, and medicinal purposes. The juice of the stem and leaves of *Gynura cusimbua* (D.Don) S.Moore is applied on fresh wounds to stop bleeding and fast healing; the leaf paste is also applied on the forehead to relieve headache and is also used for deworming and analgesic drug by the local people (21, 22). Eating of fresh and dry root of *Diplazium esculentum* (Retz.) Sw. cures dysentery (23). The sundried slices of fruits of *Garcinia cowa* Roxb. ex Choicy are used for garnishing vegetable-based curries (24). It is also the source of a natural diet ingredient hydroxycitric acid (HCA) which is an anti-obesity compound (25). *Eryngium foetidum* L. is an important spice as well as culinary herb used to garnish, marinate, flavor, and season different cuisines (26). Young leaves of *Zanthoxylum rhetsa* DC. are used as a vegetable. At the same time, fruit and stem bark are aromatic, stimulant, astringent, and prescribed for stomach aches, digestive disorders, urinary diseases, dyspepsia, diarrhea, and with honey in rheumatism (27). Local markets in the region sell *Clerodendrum glandulosum* Lindl. for various purposes, including household consumption as a vegetable stew (28); leaves are used as a therapeutic agent against diabetes, obesity, and hypertension (29–31). People from Mon district of Nagaland preferred *Herpetospermum operculatum* for soup preparation. Tender inflorescences of *Trichodesma khasianum* C.B. Clarke are cooked and chopped into pieces to make chutney with dried or fermented fish (32). Leaves and tender shoots of *P. pedicellatum* are used as vegetables by the people of Sikkim, Arunachal Pradesh, and the tribes of Manipur (33). *Litsea cubeba* Blume is used by the tribal people of the region as a spice and as medicine. Tender leaves of *Elatostemma sessile* are cooked as vegetables by the Monpa community of Arunachal Pradesh (34). There is considerable variability in the wild edible plants of the NEH region, which can be tapped for nutritional, socio-economic, ecological, and livelihood improvement of the tribal people of the region.

### 3.2. Nutritional quality

Significant variations were observed in selected wild edible plants’ nutritional quality. Results are presented (Table 1) as 3 years mean to account for temporal and spatial variations. The result of the analysis revealed that the edible part ranged between 54.1 and 98.9%, with the highest value in *Centella asiatica* (98.92%), followed by *Litsea cubeba* (83.4%) and *Herpetospermum operculatum* (82.4%). The moisture content ranged between 72.2 and 91.8%, with the highest value in *Eryngium foetidum* (91.8%) and gave significant negative correlation with most of the nutritional traits as moisture act as a diluent (Table 2). The high moisture content in leafy vegetables significantly impacts energy density (amount of energy in a given food weight). Water adds substantial weight to food without energy and provides better satiety (35). Different authors have reported similar moisture content in some wild edible plants, such as 60–68% in *Litsea cubeba* (36); 56.2, and 56.9% in *Clerodendrum* sp. and *Zanthoxylum acanthopodium*, respectively, (37); 79.3, 85.2, 82.2% in *Clerodendrum glandulosum*, *Diplazium esculentum*, and *Zanthoxylum rhetsa*, respectively, (38). An inorganic residue that reflects the entire amount of minerals in food is ash (39). A higher ash concentration predicts the existence of a variety of mineral components (40). Ash concentration ranged from 0.99 to 4.95%, with *Elatostema* sp. (4.95%) having the highest value and *Garcinia cowa* having the lowest (0.99%). The above-cited results are at par with the reported value of 6.08% ash content in *Houttuynia cordata* and 7.2% in *Zanthoxylum acanthopodium* (36). Similarly, Singh et al. (8) have also reported ash content of 0.87% in *Eryngium foetidum*, 1.91% in *Diplazium esculentum*, 2.45% in *Gynura cusimbue*, and 4.05% in *Solanum spirale.* Protein helps build and maintain whole-body tissue and forms an essential part of enzymes, fluids, and hormones. It also helps form antibodies to fight against the infection, supply energy, and build the body (41). Protein content ranged between 1.71 and 6.66%, with the highest value in *Herpetospermum operculatum* (6.66%) and the lowest in *Elatostema* sp. (1.71%). Other plants which have also depicted rich protein contents are *Trichodesma khasianum* (5.89%) and *Plukenetia corniculata* (5.27%). Protein was found to have a significant positive correlation with total phenols, antioxidant activity indicator CUPRAC, and FRAP, iron, and magnesium content (Table 2). Protein content in the range of about 6% in leaves on a fresh weight basis is also reported for *Gynura nepalensis, Pouzolzia zeylanica* and upto 9% in *Sauropus androgynus* (8). The crude protein content of 18.5 g/100 g in *Diplazium esculentum* on a dry weight basis (42). In leaves of *Centella asiatica* protein content is reported on a fresh weight basis in the range of 3–4% (43, 44). Fats represent the chemical energy and contain twice the calorific value equivalent to the molecular weight of sugar, which acts as a carrier for fat-soluble vitamins and antioxidants. The fat content as recorded in *Plukenetia corniculata* (1.36%), *Litsea cubeba* (1.18%), and *Zanthoxylum rhetsa* (0.50%) is comparable with the common vegetables like spinach (0.7%) and lettuce (0.20%) (45). The results conform with previous reports of 1.69% crude fat content in *Clerodendrum colebrookianum* and 2.91% in *Gynura cusimbua* (38). While in *Eryngium foetidum* fat content of 0.60–1.34% is reported (8, 46). Dietary fiber contributes to the bulk of food, provides satiety, reduces glycemic index, and maintains gut health. In the present analysis, dietary fiber content ranged between 5.16 and 16.0%, with the highest value in *Litsea cubeba* and the lowest in *Houttuynia cordata* (5.16%), which is almost at par with the dietary value of commercial fruits and vegetables like apple (3.2%), broad beans (8.9%), cabbage (2.8%), potato (1.7%), and spinach (2.5%) (45). The high crude fiber content (16–20%) in *Litsea cubeba* is very well reported (47). Sugar plays a vital role in carbohydrate metabolism (48). Hence, the sugar content level indicates the leaves’ supply capacity and grains’ ability to use as assimilates (49). The data of the present investigation revealed that starch content ranged between 0.27 and 2.14%, with the highest value in *Litsea cubeba* (2.14%) and the lowest in *Elatostema* sp. As per literature, several authors have reported carbohydrate and starch content in different wild edible plants, such as 3.81% carbohydrate in *Centella asiatica* (43), 5.45% in *Diplazium esculentum* (42), and 4.5% in *Eryngium foetidum* (46). Among commonly consumed green leafy vegetables amaranth and fenugreek are rich in ash, protein, fat, and dietary fiber (50), however, their value is far lower than results obtained for some wild edible plants such as *Herpetospermum operculatum, Plukenetia corniculate* and *Trichodesma khasianum.*

**TABLE 1:** Proximate composition of wild edible plants of North Eastern region, India.

Species	Edible (%)	Moisture (%)	Ash (%)	Protein (%)	Fat (%)	Dietary fiber (%)	Sugar (%)	Starch (%)
*Gynura cusimbua*	63.98	90.65	1.93	3.73	0.40	5.69	0.34	0.71
*Centella asiatica*	98.92	91.21	1.35	2.28	0.29	5.44	0.92	0.73
*Diplazium esculentum*	77.74	91.41	1.51	3.87	0.22	6.54	0.30	0.62
*Garcinia cowa*	62.12	90.64	0.99	2.75	0.41	5.55	0.42	0.96
*Eryngium foetidum*	69.28	91.81	1.03	2.51	1.47	6.18	0.83	1.09
*Zanthoxylum rhetsa*	79.75	88.14	1.82	3.64	0.50	11.15	0.67	1.11
*Houttuynia cordata*	58.49	91.72	1.23	2.41	0.36	5.16	0.56	0.89
*Clerodendrum glandulosum*	54.14	87.17	1.57	3.75	0.37	9.01	0.97	1.04
*Herpetospermum operculatum*	82.4	80.0	3.43	6.66	0.35	6.98	1.12	0.34
*Plukenetia corniculata*	73.2	72.7	4.57	5.27	1.36	11.13	2.80	0.95
*Trichodesma khasianum*	78.2	72.2	3.96	5.89	0.35	14.58	0.84	0.43
*Piper pedicellatum*	66.9	79.9	4.09	4.56	0.47	9.88	1.81	0.29
*Litsea cubeba*	83.4	72.3	1.16	3.47	1.18	16.00	3.41	2.14
*Elatostema* sp.	71.7	85.9	4.95	1.71	0.39	6.80	0.57	0.27
**Proximate composition of common green leafy vegetables Indian Food Composition Table 2017 (IFCT2017)**								
Amaranthus gangeticus (Amaranth)		86.9	2.52	3.29	0.65	4.41	0.32	0.70
Trigonella foenumgraecum (Fenugreek)		86.7	1.69	3.68	0.83	4.90	0.88	0.60
Lactuca sativa (Lettuce)		92.3	1.11	1.54	0.27	1.79	0.22	1.50
Brassica juncea (Mustard)		88.2	1.47	3.52	0.51	3.92	0.04	1.41
Spinacia oleracea (Spinach)		90.3	2.47	2.14	0.64	2.38	0.24	1.38

*n* = 3.

**TABLE 2:** Correlation between different biochemical traits.

	Ash	Protein	Fat	Dietaryfiber	Sugar	Starch	Phenol	Cuprac	Frap	Fe	Zn	Cu	Mg
Moisture	-0.589*	-0.659*	-0.354	-0.846**	-0.773**	-0.139	-0.642*	-0.678**	-0.634*	-0.474	-0.132	0.167	-0.129
Ash		0.455	-0.014	0.269	0.219	-0.597*	0.201	0.408	0.173	0.333	0.485	0.040	0.230
Protein			-0.011	0.448	0.290	-0.245	0.750**	0.678**	0.712**	0.572*	0.245	0.443	0.614*
Fat				0.345	0.652*	0.573*	0.079	0.331	0.116	0.376	0.293	-0.321	-0.214
Dietaryfiber					0.691**	0.426	0.522	0.419	0.546*	0.296	-0.210	-0.164	-0.093
Sugar						0.543*	0.523	0.701**	0.536*	0.187	0.140	-0.223	0.016
Starch							0.118	0.070	0.171	-0.153	-0.300	-0.377	-0.343
Phenol								0.842**	0.969**	0.398	0.052	0.202	0.708**
Cuprac									0.836**	0.453	0.419	0.155	0.587*
Frap										0.393	0.025	0.230	0.614*
Fe											0.371	-0.086	0.186
Zn												0.052	0.270
Cu													0.539*

*Correlation is significant at the 0.05 level (2-tailed). **Correlation is significant at the 0.01 level (2-tailed).

### 3.3. Total antioxidant activity

Phenolics are non-nutritive secondary metabolites found in plants that are beneficial in curing various diseases due to their antioxidant activity. Free radicals encourage the oxidation of proteins, DNA, and lipids, resulting in disturbances and functional loss of biological membranes and enzymes, ultimately producing toxins. Free radical scavenging is an important function of antioxidants (51–54). The wild edible plants selected for the study contain phenols ranging from 0.30 mg/g (*Elatostema* sp.) to 6.00 mg/g (*Herpetospermum operculatum*) (Table 3). The Cupric Reducing Antioxidant Capacity (CUPRAC) method is a simple and versatile antioxidant capacity assay useful for analyzing various secondary metabolites with antioxidant potential. A higher CUPRAC value indicates higher antioxidant activity. In the present study, the CUPRAC value ranged between 1.10 and 8.40 mg/g, with the highest (8.40 mg/g) recorded in *Plukenetia corniculata, Herpetospermum operculatum* and the lowest in *Diplazium esculentum* (1.10 mg/g). Ferric reducing antioxidant power (FRAP) assay was another method used to investigate the antioxidant activity of wild edible plants in this study. FRAP values ranged between 0.10 and 1.90 mg/g with the highest value in *Herpetospermum operculatum* (1.90 mg/g) and the lowest in *Elatostema* sp. (0.10 mg/g). High phenolic content (2.39 mg/g) is associated with high antioxidant activity (94.94%) inhibition of free radicals in DPPH assay (42) as tested in *Diplazium esculentum*. A highly positive correlation of phenols with CUPRAC (0.842) and FRAP (0.969) was observed in our samples, (Table 2) which is in conformity with facts and reports that phenols contribute maximum to antioxidant activity.

**TABLE 3:** Total antioxidant activity and mineral composition of wild edible plants of North Eastern region, India.

Plants	Phenol (mg/g)	Cuprac (mg/g)	Frap (mg/g)	Fe (mg/100 g)	Zn (mg/100 g)	Cu (mg/100 g)	Mg (mg/100 g)
*Gynura cusimbua*	1.00	1.90	0.30	2.84	0.59	0.34	102
*Centella asiatica*	1.70	3.40	0.60	5.30	1.25	0.19	42.6
*Diplazium esculentum*	0.80	1.10	0.30	1.86	1.49	0.40	38.6
*Garcinia cowa*	1.60	2.10	0.30	4.03	1.58	0.08	38.6
*Eryngium foetidum*	1.30	2.50	0.50	7.51	1.60	0.20	46.5
*Zanthoxylum rhetsa*	2.90	3.70	0.90	4.44	1.21	0.26	159
*Houttuynia cordata*	2.10	3.50	0.80	2.51	0.58	0.12	49.9
*Clerodendrum glandulosum*	3.50	4.90	1.50	3.31	1.04	0.38	128
*Herpetospermum operculatum*	6.00	8.00	1.90	7.19	2.39	0.34	505
*Plukenetia corniculata*	3.00	8.40	1.10	7.98	3.36	0.19	54.9
*Trichodesma khasianum*	3.40	3.60	1.20	9.40	0.52	0.17	17.3
*Piper pedicellatum*	3.70	6.20	1.10	3.70	1.09	0.37	237
*Litsea cubeba*	4.10	5.30	1.40	2.87	0.27	0.07	21.3
*Elatostema* sp.	0.30	1.50	0.10	2.31	1.99	0.09	13.4
**Total phenola and mineral profile of common green leafy vegetables (IFCT2017)**							
Amaranthus gangeticus (Amaranth)	0.238			4.64	0.86	0.21	194
Trigonella foenumgraecum (Fenugreek)	1.05			5.69	0.54	0.18	63.7
Lactuca sativa (Lettuce)	0.195			2.73	0.51	0.14	43.2
Brassica juncea (Mustard)	0.380			2.84	0.68	0.24	51.6
Spinacia oleracea (Spinach)	2.33			2.95	0.46	0.17	87.0

n = 3. Fe, Iron; Zn, Zinc; Cu, Copper; Mg, Magnesium.

### 3.4. Mineral composition

Green leafy vegetables are generally considered a rich source of minerals, and the results of the present investigation also corroborate these findings w.r.t. wild leafy vegetables (Table 3). The highest concentration of iron (Fe) was recorded in *Trichodesma khasianum* (9.40 mg/100 g) and the lowest in *Diplazium esculentum* (1.86 mg/100 g). Among commonly consumed green leafy vegetables fenugreek leaves have the highest iron content of 5.69 mg/100 g, followed by amaranth leaves having 4.64 mg/100 g, which is nearly half of maximum content obtained in *Trichodesma khasianum* (50). These plants can fulfill the daily iron requirement and prevent iron deficiency (55). Zinc (Zn) is an important mineral considered a nucleic acid metabolizer, membrane stabilizer, and immune response stimulator (56). Its concentration was between 0.27 mg/100 g (*Litsea cubeba*) and 3.36 mg/100 g (*Plukenetia corniculata*). Amararanth and brassica leaves have high content of zinc 0.86 and 0.68 mg/100 g, respectively, (50), which is much lower than the content we found in most of the wild edible plants evaluated in this study. Copper (Cu) is a vital mineral for the human body, and it serves as a cofactor for several enzymes involved in oxidation-reduction reactions and hematopoiesis (56). A significant amount of Cu was also found in the plants analyzed in the study. The concentration ranged between 0.07 and 0.40 mg/100 g, with the highest value in *Diplazium esculentum* (0.40 mg/100 g), followed by *Clerodendrum glandulosum* (0.38 mg/100 g) and *Piper pedicellatum* (0.37 mg/100 g) which are nearly double of the reported value of copper content in commonly consumed GLV, namely, mustard and amaranth leaves (50) and our findings conform with (55). Magnesium (Mg) is essential to maintain normal nerve and muscle functions. Regularly consuming Mg-rich food resources can control blood glucose levels and support a healthy immune system (55). Mg concentrations ranged between 13.4 and 505 mg/100 g, with the highest amount in *Herpetospermum operculatum* which is more than double of amaranth leaves (50). However, a good amount of Mg was also present in *Piper pedicellatum* (237 mg/100 g) and *Zanthoxylum rhetsa* (159 mg/100 g). Magnesium content of the studied wild edible plants, as shown in Table 3, are at par with some common leafy vegetables. Consumption of these locally available plants meets nutritional needs and is part of traditional food habits and customs. Our findings are in conformity with (37) for iron (1.55 mg/g), zinc (0.18 mg/g), copper (0.017 mg/g) in *Clerodendrum colebrokianum*; zinc (0.09 mg/g), iron (0.96 mg/g), copper (0.008 mg/g) in *Houttuynia cordata;* and zinc (0.867 mg/g), iron (1.175 mg/g), copper (0.012 mg/g) in *Zanthoxylum acanthopodium*. *Centella asiatica* contains iron (18 mg/100 g), magnesium (271 mg/100 g), zinc (20 mg/100 g), copper (7 mg/100 g). Similar result were also obtained by Odhav et al. (43), *Diplazium esculentum* is reported to have copper (14.38 mg/100 g), magnesium (9.56 mg/100 g), zinc (0.10 mg/100 g) as reported by Chettri (44) while *Eryngium foetidum contains* magnesium (98.5 mg/100 g), iron (111.21 mg/100 g), zinc (4.5 mg/100 g), copper (441.4 mg/100 g) as reported (57, 58).

## 4. Conclusion

The present article is an attempt to know the nutritional contribution, supplementary role, and market potential of wild edibles to boost the economy and nutritional security of traditional communities of the NEH region. NEH region being a hotspot of biodiversity, thorough investigation for validation of the nutritional potential of WEPs is utmost required. Given the conservation and popularization as future potential resources, a lot of issues about their domestication, selection of quality material, development of good cultivation practices, and value addition for wider acceptability are required to be addressed. Considerable variations existed in the nutritional value, total antioxidant activity, and mineral composition of wild edible plants selected for nutritional profiling under the present study. Most wild edible plants exhibited higher concentrations of nutrients such as ash, protein, fat, dietary fiber, sugar, starch, phenol, antioxidants, iron, zinc, copper, and magnesium than commercial crops. *Herpetospermum operculatum, Plukenetia corniculata, Trichodesma khasianum*, and *Piper pedicellatum* are the most nutritious wild leafy vegetables, with high proportions of protein, ash, sugar, starch, total phenols, antioxidant activity, iron, zinc, and magnesium, according to overall nutritional profiling. In Nagaland, *Herpetospermum operculatum* and *Plukenetia corniculata* are in high demand, and commercial production of these plants has begun in some areas to meet demand. Similarly, *Clerodendron glandulosum* and *Spilanthes accmella* are also much sought after locally for their food and medicinal value. As a result, it can be inferred that wild edible plants are nutrient-dense and can be used as an alternative source of nutrients to help alleviate poverty and achieve food and nutritional security. Domestication and promotion of commercial cultivation of these wild edible plants will generate substantial income for the poor farmers residing in the region’s remote areas. Moreover, immediate efforts for collecting their diverse germplasm for *ex situ* conservation are equally important at the national level. These wild leafy vegetables can be promoted in the region through their inclusion in the All India Coordinated Network Project on Potential Crops.

## Data availability statement

The original contributions presented in this study are included in the article/Supplementary material, further inquiries can be directed to the corresponding authors.

## Author contributions

HT, AY, RR, KP, SL, BI, LS, RA, MD, and V: collection of samples from Meghalaya, Nagaland. HT: preparation of draft manuscript. AY, HT, and RB: biochemical evaluation. SA and KB: documentation of traditional knowledge. AK: formal analysis. KB and RB: critical review. All authors contributed to the article and approved the submitted version.
