# Supplementary figures and images for: Nutritional evaluation of some potential wild edible plants of North Eastern region of India
Source: Front Nutr. 2023 Mar 1;10:1052086. doi: 10.3389/fnut.2023.1052086 (PMC10014872; doi:10.3389/fnut.2023.1052086)

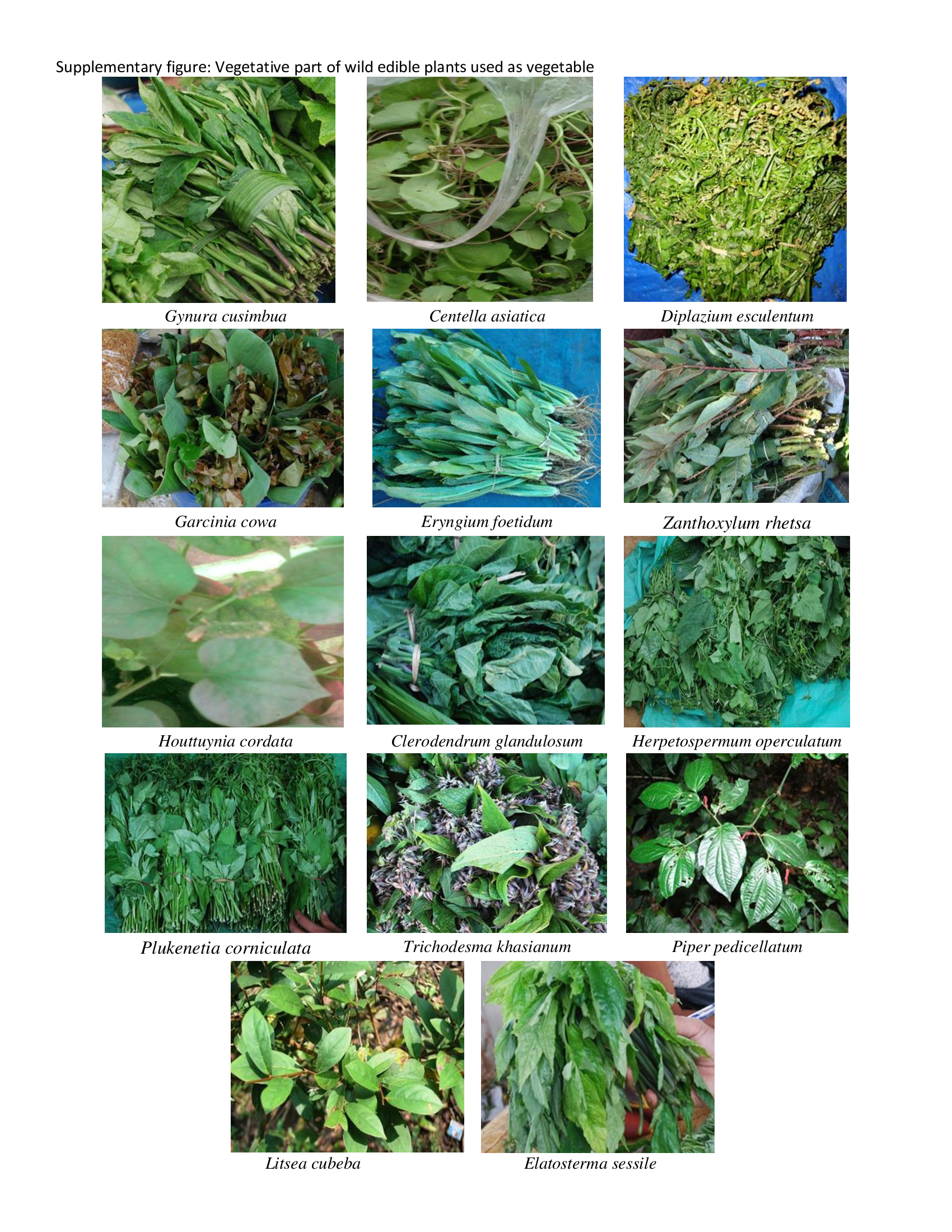

Supplement: Supplementary file 1 [file Image_1.JPEG]
