# Supplementary material for: Nutritional evaluation of some potential wild edible plants of North Eastern region of India
Source: Front Nutr. 2023 Mar 1;10:1052086. doi: 10.3389/fnut.2023.1052086 (PMC10014872; doi:10.3389/fnut.2023.1052086)
Supplement: Supplementary file 2 [file Table_1.docx]

**Supplementary Table: Wild edible plants used by indigenous population of North Eastern Region**

| **Species** | **Family** | **Local name** | **Habit** | **Habitat** | **Flowering/**  **Fruiting** | **Edible part** | **Characteristics and Uses** |
| --- | --- | --- | --- | --- | --- | --- | --- |
| *Gynura cusimbua* (D.Don) S.Moore | Asteraceae |  | Herb | Moist forest of Khasi and Jaintia Hills, and Sikkim; cultivated in Nagaland | January-March | Fruit, Leaves | Commonly found in roadsides, fields, and grassy slopes. Leaves are used for soup preparation and boiled along with leaves/young shoots of *Spilanthus oleracaea*, *Zanthoxylum rheta* as a vegetable [1]. Sometimes leaves are also dried, and paste is made for consumption. The juice of stem and leaves is applied on fresh wounds to stop bleeding and fast healing. The leaf paste is also applied on the forehead to relieve headaches and is used as a sedative drug by the local people [2-3]. |
| *Centella asiatica* (L.) Urb. | Apiaceae |  | Herb | Throughout the region in disturbed moist areas | - | Leaves | It is either used as salad alone or combined with onions, crushed peanuts, and bean powder and seasoned with lime juice and fish sauce. It is also widely used as chutney along with dried red chili and fermented fish called "tungtap". Besides culinary uses, leaves are supposed to be useful to improve memory, cure skin diseases, syphilis and rheumatism [4]. |
| *Diplazium esculentum* (Retz.) Sw. | Athyriaceae | Muikhun chak – Tripura  Rukja/Horon – Arunachal Pradesh  Dhekia/Takan – Assam  Cha-Kawk – Mizoram  Lai-changkhrang – Manipur  Kongtongshik – Nagaland  Jhur tyrkhang -Meghalaya, ningro/ Jilmilsag-Sikkim | Forb/herb | Exposed or in partial shade in disturbed places, in wet areas such as along stream banks | Throughout the year | Fronds | It is a terrestrial fern, perennial in nature,  It grows in moist and shady places of wasteland and forest etc. Usually available for consumption from March to December [5]. Tender fern crochets are used as green vegetables and as a salad or cooked as vegetables [6]. Matured leaves (dried fronds) are used as cattle bedding material from June to October/November. Besides use as vegetable, decoction is used to cure haemoptysis and cough. Further, eating fresh and dry root cures dysentery [7]. |
| *Garcinia cowa* Roxb. ex Choicy | Clusiaceae |  | Tree | Frequent in evergreen and mixed forest at lower elevations | March-August | Fruit | It is one of the famous indigenous fruits of the NEH region of India. The fruit has commercial and medicinal values. The sundried slices of its fruits are used to garnish vegetable-based curries [8]. It is the source of a natural diet ingredient hydroxycitric acid (HCA), an anti-obesity compound [9]. |
| *Eryngium foetidum* L. | Apiaceae | Adi Dhania | Herb | Along forest margins; also in kitchen gardens | May-February | Leaves | It is an important spice-cum culinary herb used to garnish, marinate, flavor, and season different cuisines [10]. Demand for this herb among ethnic populations of the region is increasing because its unique pungent aroma gives the characteristic flavor to the dishes [11]. This herb is considered an alternative to coriander due to its similar aroma [12, 13]. Besides, it has been used as traditional ethnomedicine for the treatment of number of ailments such as fevers, chills, vomiting, burns, earache, fevers, hypertension, headache, constipation, fits, asthma, stomachache, arthritis, worms, infertility complications, snake bites, scorpion stings, diarrhea, malaria and epilepsy [14, 15]. |
| *Zanthoxylum rhetsa* DC. | Rutaceae | Onger | Tree | Common in secondary forests of the region | September-April | Fruit, Leaves | It is widely distributed in warm, temperate and subtropical areas worldwide. It is found in Assam, Meghalaya, and the eastern Western Ghats of peninsular India [16]. Its young leaves are used as a vegetable. The fruit and stem bark are aromatic, stimulant, astringent, stomachic, and digestive; prescribed in urinary diseases, dyspepsia, diarrhea, and honey in rheumatism [17]. |
| Houttuynia cordata Thunb. | Saururaceae | Jamyrdoh | Herb | Mostly in open places and along streams | April-July | Leaves | It is an aromatic medicinal herb with creeping rootstock [18]. The root, young shoots, leaves, and sometimes the whole plant is traditionally used as salad throughout the region [19]. Besides, the shoot has been used for freshness, good sleep, heart disorders by the Apatani tribe of Lower Subansiri district of Arunachal Pradesh [20]. |
| *Clerodendrum glandulosum* Lindl. | Verbenaceae | Jarem | Shrub | Khasi and Garo hills of Meghalaya; throughout NE India; also cultivated | February-August | Leaves | This plant has a long association with the culture and the tradition of people of NEH region since early 16^th^ century. The plant is used as an ingredients in the preparation of a special menu called 'uti' a naturally carbonated mixed vegetable pourice, served during the New Year day in Manipur calendar locally known as "Sajibu cheiraoba". It is believed that, one taking uti on the first day of a year will endure long without any ailments until the end of the year [21]. Local markets in the region sell this plant for various purposes, including household consumption as a vegetable stew [22]. Leaves are used as a therapeutic agent against diabetes, obesity, and hypertension (20, 23, 24]. |
| Herpetospermum *operculatum* K.Pradheep, A.Pandey, K.C.Bhatt & E.R.Nayar | Cucurbitaceae | Chi patta | Twining climber | Common among thickets and along riverbanks at altitudes of 1500–3000 m | August-October | Leaves | It is locally known as Thruinam (Mon) and found to occur in North-eastern India (Nagaland, Sikkim), Myanmar (Kachin), and China (western Yunnan, southeast Xizang). It grows in little disturbed subtropical to temperate forests, in thickets, over bushes and small trees, upon rock, in open situations, along riverbanks up to an altitude of 1500–2500 m [22]. This plant is preferred by Mon people of Nagaland for soup preparation. |
| *Plukenetia corniculata* Sm. | Euphorbiaceae | Meetha patta | Vine | Disturbed areas of tropical wet and moist forests at low and medium altitudes (50–800 m). | July-Sept/Oct-Nov | Leaves | Locally known as 'meetha patta' and used as leafy vegetables. Young shoots have high protein and ascorbic acid levels of 5.6 % and 643 mg/100 g [25] on a fresh weight basis, respectively. Tribal people of Nagaland used this as vegetable and soup-making, apart from delicious curry prepared with it. Tender shoots and leaves are also consumed by the tribes in the Diphu area of Assam, but to a lesser extent, through boiling with daal (pulse) and other leafy vegetables [26]. |
| *Trichodesma khasianum* C.B. Clarke | Boraginaceae | Euthepe | Herb | Wet places, sand dunes, wasteland, and hedges | October-November | Leaves | It is a small tree, up to 5 m tall, commonly found in Arunachal Pradesh, Assam, and Manipur. Tender inflorescences are cooked and chopped into pieces to make chutney with dried or fermented fish [27]. |
| *Piper pedicellatum* C. DC. | Piperaceae | Rori | Shrub | Subtropical and warm broad-leaved forests. | October - February | Leaves | It grows as undergrowth and spreads on the ground, as a shrub, or as climbers on shrubs and trees. Leaves and tender shoots of *P. pedicellatum* are used as vegetables by the people of Sikkim, Arunachal Pradesh, and the tribes of Manipur [28]. Besides culinary use, it has antifungal and antioxidant activity [29], while the tribal people of the region use its young shoots as medicines to relieve internal body pain [30]. |
| *Litsea cubeba* Blume | Lauraceae | *Tenga Patta* | Tree | Semi-shade or no shade in moist soil. | - | Fruit, Bark | The tribal people of the NEH region use it as a spice and as medicine. Apatani people of Arunachal Pradesh use both ripe and unripe fruits as substitutes for spices and used in preparation for vegetables, curry, and meat and pickles [31]. Similarly, people in the Lakhimpur district of Assam use its bark to treat pneumonia or tuberculosis [32]. |
| *Elatostemma* sessile | Urticaceae |  | Herb | Dark, damp places, amongst rocks or on banks of underground streams at the base of a large limestone cave | July-Aug. / Sept - Nov. | Leaves | *Elatostema cuneatum*, *E. laetevirens*, *E. lineolatum*, *E. sessile,* locally known as Chulukpa, Makino, Dambe-hru, and Fambe-che, respectively are commonly found in the NEH region. Tender leaves are cooked as vegetables by the Monpa community of Arunachal Pradesh [33]. |

**References for Supplementary Table**

1. Rana VS, Blazquez MA. Chemical constituents of Gynura cusimbua aerial parts. Journal of Essential Oil Research. 2007 Jan 1;19(1):21-2.
2. Sinha SC. Medicinal plants of Manipur. 1996. p. 84. Mass and Sinha Pub., Manipur.
3. Devi LD. Folklore on the use of Indigenous plants and Animals in Manipur Vol. 2. L, D, Devi,[mphal. 1998.
4. Devkota A, Jha PK. Biology and medicinal characteristics of Centella asiatica. Medicinal plants in Nepal: an anthology of contemporary research. 2008:68-80.
5. Pradhan S. and Tamang, JP. Ethnobiology of wild leafy vegetables of Sikkim. *Indian Journal of Traditional Knowledge*, 2015; 14 (2):2090-297.
6. Upreti K, Jalal JS, Tewari LM, Joshi GC, Pangtey YP, Tewari G. Ethnomedicinal uses of Pteridophytes of Kumaun Himalaya, Uttarakhand, India. J Am Sci. 2009;5(4):167-70.
7. Sundriyal M, Rai LK. Wild edible plants of Sikkim Himalaya. J Hill Res. 1996;9(2):267-78.
8. Dutta S, Neog M, Saikia A. Shelf-life enhancement of cowa (Garcinia cowaRoxb.). International Journal of Processing and Post Harvest Technology. 2017;8(1):50-5.
9. Jena BS, Jayaprakasha GK, Sakariah KK. Organic acids from leaves, fruits, and rinds of Garcinia cowa. Journal of Agricultural and Food chemistry. 2002 Jun 5;50(12):3431-4.
10. Singh BK, Ramakrishna Y, Ngachan SV. Spiny coriander (Eryngium foetidum L.): a commonly used, neglected spicing-culinary herb of Mizoram, India. Genetic Resources and Crop Evolution. 2014 Aug;61(6):1085-90.
11. Boonsong E. *Seed development and umbel order contribution on eryngio (Eryngium foetidum L.) seed yield and quality* (Doctoral dissertation, PhD Thesis, The Graduate School, Kasetsart University, Chatuchak, Bangkok 10903, Thailand).
12. Seaforth C, Tikasingh T, Gupta M, St Rose G. A study for the development of a handbook of selected Caribbean herbs for industry.
13. Chowdhury JU, Nandi NC, Yusuf M. Chemical constituents of essential oil of the leaves of Eryngium foetidum from Bangladesh. Bangladesh Journal of Scientific and Industrial Research. 2007;42(3):347-52.
14. Roumy V, Garcia-Pizango G, Gutierrez-Choquevilca AL, Ruiz L, Jullian V, Winterton P, Fabre N, Moulis C, Valentin A. Amazonian plants from Peru used by Quechua and Mestizo to treat malaria with evaluation of their activity. Journal of ethnopharmacology. 2007 Jul 25;112(3):482-9.
15. Paul JH, Seaforth CE, Tikasingh T. Eryngium foetidum L.: A review. Fitoterapia. 2011 Apr 1;82(3):302-8.
16. Antony P, Nagaveni MA, Tallur PN, Naik, VM. Zanthoxylum rhetsa DC, a potential antioxidant preservative to control the rancidity of peanut oil. *Journal of Ultra Chemistry*, 2019;15(2), 9-17.
17. Rastogi R, Mehrotra B. Compendium of Indian Medicinal Plants published by Central Drug Research Institute. Lucknow and National Institute of Sciences Communication and Information Resources, New Delhi. 1990;1994(6):395-8.
18. Bhattacharyya N, Sarma S. Assessment of availability, ecological feature, and habitat preference of the medicinal herb Houttuynia cordata Thunb in the Brahmaputra Valley of Assam, India. Environmental monitoring and assessment. 2010 Jan;160(1):277-87.
19. Jiangang F, Ling D, Zhang L, Hongmei L. Houttuynia cordata Thunb: a review of phytochemistry and pharmacology and quality control. Chinese Medicine. 2013 Aug 21;2013.
20. Kala CP. Ethnomedicinal botany of the Apatani in the Eastern Himalayan region of India. Journal of ethnobiology and Ethnomedicine. 2005 Dec;1(1):1-8.
21. Jadeja RN, Thounaojam MC, Singh TB, Devkar RV, Ramachandran AV. Traditional uses, phytochemistry and pharmacology of Clerodendron glandulosum Coleb–a review. Asian Pacific Journal of Tropical Medicine. 2012 Jan 1;5(1):1-6.
22. Pradheep K, Pandey A, Bhatt KC. Wild edible plants used by Konyak tribe in Mon district of Nagaland: Survey and inventorisation. Indian Journal of Natural Products and Resources (IJNPR)[Formerly Natural Product Radiance (NPR)]. 2016 Apr 21;7(1):74-81.

Kala, C. P. 2005. Ethnomedicinal Botany of the Apatani in the Eastern Himalayan Region of India. *Journal of Ethno- biology and Ethnomedicine*, 1(11):11-18.

1. Sourabh D, Arunachalam A, Das AK. Indigenous knowledge of Nyishi tribes on traditional agroforestry systems. Indian Journal of Traditional Knowledge. 2009;8(1):41-6.
2. Jadeja R, Thounaojam M, Ramachandran AV, Devkar R. Phytochemical constituents and free radical scavenging activity of Clerodendron glandulosum. coleb methanolic extract. Journal of Complementary and Integrative Medicine. 2009 Jul 10;6(1).

Pradheep, K., Soyimchiten, Pandey, A. and Bhatt, K C. 2016. Wild edible plants used by Konyak tribe in Mon district of Nagaland: Survey and inventorisation. *Indian Journal of Natural Products and Resources*, 7(1): 74-81.

1. Krishnamurthi A. The Wealth of India: Raw Materials: Vol. VIII. Ph-Re. The Wealth of India: Raw Materials: Vol. VIII. Ph-Re.. 1969.
2. Pradheep K, Rathi RS, Nayar ER. “Meetha patta”(Plukenetia corniculata Sm.): a new report of leafy vegetable crop from north-eastern region of India. Genetic resources and crop evolution. 2015 Oct;62(7):1113-20.
3. Pfoze NL. *Ethnobotanical studies and phytochemical analysis of selected medicinal plants of Senapati district Manipur* 2012 (Doctoral dissertation, North Eastern Hill University Shillong).
4. Chanchal C, Thongam B, Handique PJ. Morphological diversity and characterization of some of the wild Piper species of North East India. Genetic Resources and Crop Evolution. 2015 Feb;62(2):303-13.
5. Tamuly C, Dutta PP, Bordoloi M, Bora J. Antifungal and antioxidant pyrrole derivative from Piper pedicellatum. Natural Product Communications. 2013 Oct;8(10):1934578X1300801029.
6. Gajurel PR, Rethy P, Kumar Y, Singh B. Piper species (Piperaceae) of North East India (Arunachal Pradesh). 2008. Bishen Singh Mahendra Pal Singh, Dehra Dun, India.
7. Srivastava RC, Singh RK, Mukherjee TK. Indigenous biodiversity of Apatani plateau: Learning on biocultural knowledge of Apatani tribe of Arunachal Pradesh for sustainable livelihoods.
8. Gogoi P. Ethnobotanical Study of Certain Medicinal Plants used by local peoplein Lakhimpur District of Assam, India. International journal of Chemtech Research. 2017;10(9):7-13.
9. Tsering J, Gogoi BJ, Hui PK, Tam N, Tag H. Ethnobotanical appraisal on wild edible plants used by the Monpa community of Arunachal Pradesh.
